# Band gap engineered zinc oxide nanostructures *via* a sol–gel synthesis of solvent driven shape-controlled crystal growth†

**DOI:** 10.1039/c9ra02091h

**Published:** 2019-05-10

**Authors:** Klinton Davis, Ryan Yarbrough, Michael Froeschle, Jamel White, Hemali Rathnayake

**Affiliations:** Department of Nanoscience, Joint School of Nanoscience & Nanoengineering, University of North Carolina at Greensboro Greensboro NC 27401 USA hprathna@uncg.edu +1-336-285-2860

## Abstract

A reliable sol–gel approach, which combines the formation of ZnO nanocrystals and a solvent driven, shape-controlled, crystal-growth process to form well-organized ZnO nanostructures at low temperature is presented. The sol of ZnO nanocrystals showed shape-controlled crystal growth with respect to the solvent type, resulting in either nanorods, nanoparticles, or nanoslates. The solvothermal process, along with the solvent polarity facilitate the shape-controlled crystal growth process, augmenting the concept of a selective adhesion of solvents onto crystal facets and controlling the final shape of the nanostructures. The XRD traces and XPS spectra support the concept of selective adhesion of solvents onto crystal facets that leads to yield different ZnO morphologies. The shift in optical absorption maxima from 332 nm in initial precursor solution, to 347 nm for ZnO nanocrystals sol, and finally to 375 nm for ZnO nanorods, evidenced the gradual growth and ripening of nanocrystals to dimensional nanostructures. The engineered optical band gaps of ZnO nanostructures are found to be ranged from 3.10 eV to 3.37 eV with respect to the ZnO nanostructures formed in different solvent systems. The theoretical band gaps computed from the experimental XRD spectral traces lie within the range of the optical band gaps obtained from UV-visible spectra of ZnO nanostructures. The spin-casted thin film of ZnO nanorods prepared in DMF exhibits the electrical conductivity of 1.14 × 10^−3^ S cm^−1^, which is nearly one order of magnitude higher than the electrical conductivity of ZnO nanoparticles formed in hydroquinone and ZnO sols. The possibility of engineering the band gap and electrical properties of ZnO at nanoscale utilizing an aqueous-based wet chemical synthesis process presented here is simple, versatile, and environmentally friendly, and thus may applicable for making other types of band-gap engineered metal oxide nanostructures with shape-controlled morphologies and optoelectrical properties.

## Abstract 2

A reliable and simple sol–gel synthesis followed by a solvent-driven, shape controlled, crystal growth process to make ZnO nanostructures is demonstrated.

## Graphical abstract

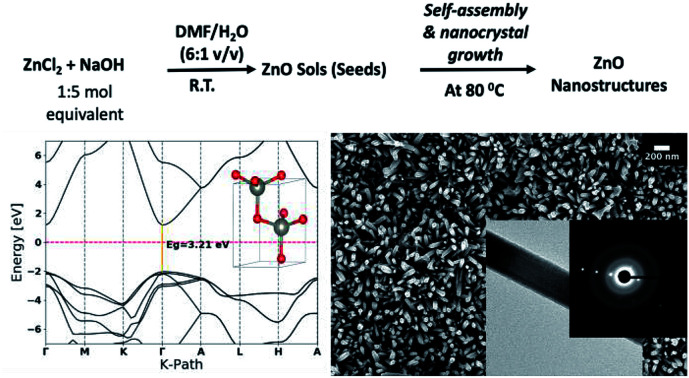

## Introduction

Zinc oxide nanostructures are functional materials that can tailor their morphology through a variety of synthesis methods to yield a wide range of morphologies such as nanowires,^1,2^ nanorods,^3–5^ nanobelts,^6^ nanocombs,^7^ nanorings,^8^ and nanocages.^9^ Owing to the lack of a centre-of-symmetry in the wurtzite crystal structure, and high exciton binding energy (60 meV), nanostructured ZnO possess strong piezoelectric^10^ and pyroelectric^11^ properties and acts as a wide band gap (3.37 eV) semiconductor for short wavelength optoelectronic devices.^12^ In recent research advancements, ZnO has received considerable attention for solar cells,^12^ lasers,^13^ spintronics,^14^ transparent conductive oxide,^12^ catalysis,^15^ bioimaging,^16^ and biosensors.^17^

Among dimensional ZnO nanomaterials, one-dimensional (1D) ZnO nanostructures with defect free high crystallinity are a particular interest due to their unique and inherent intrinsic chemical, electrical, physical, and mechanical properties compared to that of bulk and thin film counterpart.^10^ However, synthesis of defect free 1D ZnO nanostructures, with desired morphology and composition, has been challenging as most growth techniques involve either high-cost fabrication processes or high temperature wet-chemical syntheses performed in highly toxic solvents. Among such fabrication techniques, chemical vapor deposition (CVD),^18^ pulse-laser deposition (PLD),^19^ molecular beam epitaxy (MBE),^20^ and electro-chemical deposition^21^ have been utilized to grow ZnO nanostructures directly onto the substrate. In addition to these thin film deposition techniques, sol–gel and solvothermal methods are two of the most common chemical solution methods, which show the promise in terms of scalability, energy efficiency, and cost-effectiveness for the preparation of catalyst-free metal oxide nanostructures with better control over the growth conditions and morphology.^22–24^ Particularly, the sol–gel process has been extensively investigated for making homogenous, highly stoichiometric, and high-quality metal oxide nanostructures such as nanorods,^25,26^ nanoflakes,^27^ nanotubes,^25,28^ and nanofibers.^29^ In general, the sol–gel process involves formation of sol from homogeneously mixed solutions of a metal precursor and a base. The sol of metal oxide nanocrystals can either be deposited onto a substrate to grow nanostructures or be continued through the polycondensation process to form gels. The gel can be used to form particles, xerogels, aerogels, glass, and ceramics, depending upon the final processing step involved.^30^

Up to date, the sol–gel method of either aqueous or non-aqueous hydrolysis and condensation process, has been adapted primarily to make sols of ZnO nanoparticles, followed by casting on substrates to grow ZnO nanostructures, upon subjecting to thermal annealing.^1,4,5,31,32^ However, as per our knowledge, there is no records of a sol–gel based green synthesis method, which combines the formation of sols and a solvent-driven shape-controlled crystal growth process in solution at low temperature to make ZnO nanostructures, with the wurtzite crystal lattice. This method benefits to make ZnO nanomaterials in powder form for solution processable thin films fabrications in large scale. Thus, herein, we demonstrate a reliable sol–gel approach, which combines the formation of a ZnO nanocrystals sol and a solvent driven shape-controlled *in situ* crystal growth process to form well-organized ZnO nanostructures at low temperature (<80 °C) in solution. This one-pot synthesis process allows us to make ZnO nanostructures with a variety of morphologies, without disturbing their crystalline structures and compositions. The ZnO nanostructures prepared in this manner showed excellent improvement in crystallinity and optoelectrical properties, with engineered optical band gap. The method developed here is a “green synthesis” where we utilized environmentally friendly and benign materials, with energy efficient wet-chemical approach. Thus, our method is rather advantages in terms of scalability, processability, and reliability compared to other wet-chemical and electrochemical synthesis methods, which typically grow nanostructures directly from thin film casted sol in environmentally friendly manner or high temperature solvothermal methods that utilize highly toxic hydrazine-based solvents and additives.

## Results and discussion

In colloidal solutions, shape-controlled nanocrystal growth is governed by: (1) the classical crystal growth kinetics of Ostwald ripening theory and the “oriented attachment” mechanism where a sol of nanocrystals with shared crystallographic orientations directly combine together to form larger ones;^33–35^ (2) the relative surface energy of crystal facets and selective adhesion of solvents/surfactants onto crystal facets; and (3) the crystal growth regime, which depends on the monomer concentration and temperature.^23,24^ Past studies demonstrated that shape-controlled crystal growth has been successfully implemented to make metal oxide nanostructures by adjusting experimental conditions, such as monomer concentration, catalysts, and solvent type, in combination with a suitable growth temperature.^36^ For example, utilizing the concept of crystal growth-oriented attachment mechanism, the formation of ZnO nanorods from ∼3 nm nanocrystals upon prolong heating was demonstrated.^37^ Also, the concept of selective adhesion of surfactants onto crystal facets has been widely tested for the shaped-controlled synthesis of a variety of metal oxide nanostructures.^38–40^ Despite the work describes herein, up to date, there has been no efficient, wet-chemical synthesis process developed, augmenting the shape-controlled crystal growth by combining Ostwald ripening and oriented attachment mechanisms. In our method, the monomer concentration and the reaction temperature were kept constant in order to minimize the effect of the crystal growth regime, which is a balance between the kinetic and thermodynamic growth regimes. Herein, we hypothesize that the polarity of the solvent selectively adsorbs onto surfaces of the growing crystallites to modulate the surface energy of nanocrystals and yields the final shape of the nanostructures.

The ZnO nanostructures were prepared by combining the sol–gel chemical process with a solvent-driven nanocrystals growth at low temperature. The typical sol–gel process involved base catalysed hydrolysis of the metal salt to form metal hydroxide followed by condensation to form nanocrystals of the metal oxide sol. Upon subjecting the sol of nanocrystals to an *in situ* solvothermal process, ZnO nanostructures with different morphologies were obtained. The solvothermal process induces the nanocrystal elongation along the facet of high energy crystal lattice surface and initiates nanocrystals growth through Ostwald ripening and oriented attachment mechanisms.^35^ Also, the highest packing efficiency of nanocrystals into well-ordered nanostructures is feasible *via* solvent-driven self-assembly of nanocrystals through van der Waals interactions.^41–44^ Such non-covalent interactions enable interacting tiny nanocrystals to form larger crystal aggregates and induce the crystal growth mechanisms (Ostwald ripening and “oriented attachment”).^34–36^ As a result, nanocrystals with sharing crystallographic orientations directly combine together to form larger ones along the most preferential crystal lattice axis. The dimensionality and morphology of these crystalline nanostructures were tailored by changing the solvent mixture. The difference in polarity and surface adhesion of each organic solvent controlled the shape and size of nanocrystal growth. The solvent molecules act as surfactants that adsorb onto surfaces of the growing crystallites.^45^ The selective adhesion of solvent molecules onto crystal lattice facets is governed by the surface energy difference, which drives solvent binding onto a selected facet. For example, past literature evidences that if a solvent has high binding affinity to the {001} facet of the crystal lattice, the growth rate along the direction of the [001] plane reduces and consequently, results in the formation of nanorods.^37,40,45^ Similarly, in our studies, we speculate that depending on the polarity, chemical functionality, hydrophilicity and hydrophobicity, solvents selectively adsorb onto different faces of the nanocrystal lattice, reducing the growth rate along a particular lattice facet to form a shape-controlled nanostructure.^40^

### Preparation and characterization

**Scheme 1:**
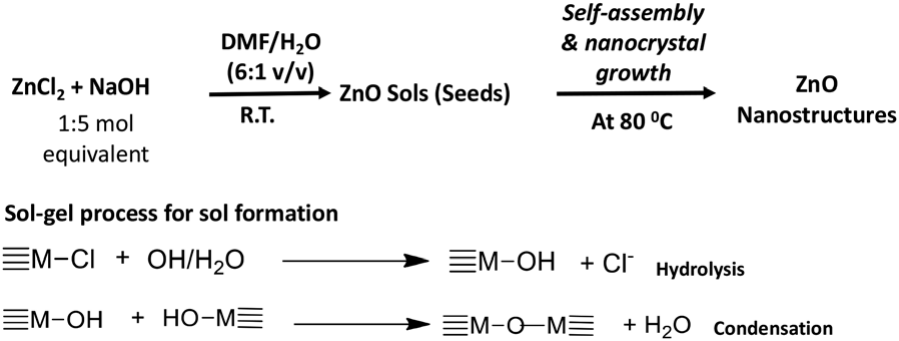
The formation of ZnO nanostructures *via* sol–gel process followed by solvothermal self-assembly.

In a typical procedure, the preparation of zinc oxide nanostructures was performed by a base-catalysed hydrolysis and condensation of zinc(ii)chloride with NaOH in a series of organic solvent–water systems in open atmosphere. The molar ratio of the metal precursor to NaOH and the nanocrystals growth temperature were tailored to achieve highly crystalline nanostructures. The chemistry of making ZnO nanostructures is depicted in Scheme 1.

**Fig. 1:**
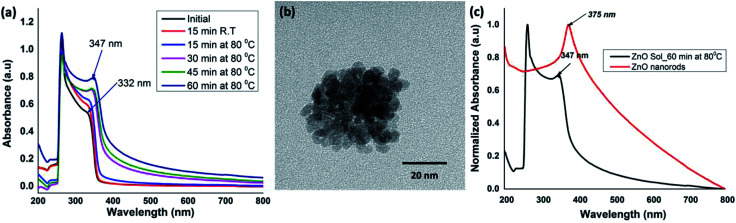
(a) Time-dependent UV-visible absorption spectra acquired during ZnO nanocrystals formation; (b) the TEM image of ZnO nanocrystals formed after heating for 60 minutes; and (c) UV-visible spectra of ZnO sols and ZnO nanorods taken after 60 min and 24 h at 80 °C respectively (all spectra were acquired in aqueous solutions).

The sol of ZnO nanocrystals was prepared by mixing 1 : 5 molar ratio of the metal precursor (ZnCl_2_) and the base (NaOH) at room temperature with initially stirring for ∼15 minutes. After ∼15 minutes of stirring at room temperature, the sol of nanocrystal formation was monitored at 15 minutes time intervals up to 60 minutes while homogeneous stirring at 80 °C to yield an opaque solution. The reaction time for the formation of ZnO sol was monitored by acquiring UV-visible spectra of the solution at different time intervals. The time-dependent UV-visible spectra were compared with the absorption traces collected for the initial reactants' solution at room temperature. All the spectra were recorded in solution as either a clear or an opaque solution. As depicted in Fig. 1(a), the UV-visible spectrum, obtained for the reactants' solution after 15 minutes of stirring at room temperature, exhibits the absorption maximum at 264 nm with a shoulder peak at 332 nm, characterizing the Zn^2+^ absorption. As the reaction progressed, a gradual red shift in the shoulder absorption band at 332 nm was observed. The reaction mixture heated for 60 minutes showed a red-shifted absorption maximum at 347 nm, evidencing the formation of ZnO nanocrystals (Fig. 1(a)). The transmission electron microscopy (TEM) images further confirmed that nanocrystals were somewhat spherical in shape with the size ranging from 3–5 nm (Fig. 1(b)).

The absorption peak in the range of 345–450 nm arises due to the surface plasma resonance effect of ZnO nanocrystals and the peak shift from blue to red is characteristic to the quantum size effect.^46–48^ The stronger exciton effect is characteristic of the quantum confinement in semiconducting nanostructures, in which the electrons, holes, and excitons have limited space to move; and their movement only is possible for definite values of energies. As a result, the continuum of states in conduction and valence bands exhibits discrete states with an energy spacing relative to band edges; which is inversely proportional to the square of the particle size and reduced mass.^48^ Thus, their energy spectrum is quantized. With the increase in crystallites size, a red shift in the absorption spectra can be observed because of narrowing the band gap, which leads to the effective band gap smaller than its bulk value. In our case, the absorption peak shift from 332 nm in initial precursor solution, to 347 nm for ZnO nanocrystals sol, and finally to 375 nm for ZnO nanorods (Fig. 1(a) and (c)) evidence the gradual growth and ripening of nanocrystals to dimensional nanostructures. As a result, we observed a gradual decrease in the band gap from 3.57 eV to 3.20 eV, confirming the presence of highly confined carriers in ZnO nanocrystals compared to that of in solvothermal grown ZnO nanorods. This is in good agreement with the previously reported results.^48^

The sol prepared in this manner was subjected to *in situ* condensation and shape-controlled nanocrystal growth process by transferring the reaction mixture onto a sand bath with no stirring while maintaining the temperature at 80 °C for overnight (24 h). The nanostructures' formation, morphologies, and crystallinities were characterized by acquiring UV-visible spectra, SEM images, and X-ray powder diffraction (XRD) traces combined with selective area electron diffraction (SAED) patterns. The atomic compositions and binding energies of Zn, O, and C present in ZnO nanostructures with respect to different morphologies were analysed using X-ray photoelectron spectroscopy.

### Morphology analysis

**Fig. 2:**
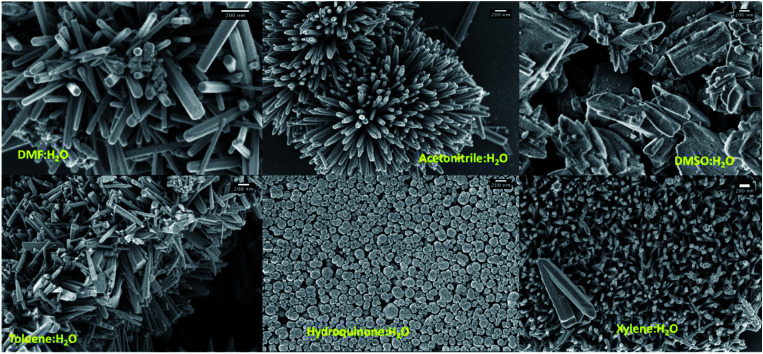
SEM images of ZnO nanostructures prepared using different solvent systems; scale bars represent 200 nm.

As shown in Table 1, shape-controlled ZnO nanostructures with different morphologies were obtained by varying the organic solvent type in the reaction mixture to six different organic solvents (dimethyl formamide, acetonitrile, dimethyl sulfoxide, toluene, hydroquinone, and xylene). The SEM analysis of the final products resulted from different solvent systems exhibit three different distinct morphologies: hexagonal nanorods, slates like structures, and globular shaped nanoparticles (see Fig. 2 and S1†). The ZnO hexagonal nanorods, with 1–5 μm in length and 50 nm to 180 nm in width were resulted in water and dimethyl formamide (DMF) mixture whereas irregular slates like structures with an average length ranged from 500 nm to 2 μm were formed in the solvent mixture of water and dimethyl sulfoxide (DMSO). Morphologies of ZnO nanostructures, resulted from solvent systems of xylene, toluene, and acetonitrile mixed with water, were shorter nanorods comparing to those resulted in from the DMF/water solvent system. Particularly, comparing to the dimensions of ZnO nanorods formed in toluene and acetonitrile mixtures, lengths of nanorods formed in *m*-xylene were considerably shorter. This may be due to the differences in solubility of each solvent in water as toluene and acetonitrile have higher solubility compared to *m*-xylene. Thus, *m*-xylene/water system has high concentration of non-solvated xylene molecules, which could hinder nanorods growth along the [001] direction due to solvent molecule adhesion, yielding shorter and smaller in diameter nanorods compared to the nanorods formed in toluene and acetonitrile. Surprisingly, the nanostructures prepared in a mixture of hydroquinone and water were globular shaped particles with an average diameter ranging from 100 nm to 500 nm.

**Table 1:** The morphologies of ZnO nanostructures obtained in different solvent systems^a^

Trial #	Solvent system (6 : 1 v/v)	Nanostructures morphology and dimension description
1	DMF : H_2_O	Hexagonal rods with the average length of 1–5 μm and width of 50–180 nm
2	Acetonitrile : H_2_O	Hexagonal rods with the average length of 1–3 μm and width of 50–180 nm
3	DMSO : H_2_O	Thin slates like structures ranging from 500–2 μm
4	Toluene : H_2_O	A mix of thin hexagonal rods and wide slates. The average length of rods 1–5 μm and width of 100–200 nm
5	Hydroquinone : H_2_O	Globular shaped particle-like structures ranging from 100–500 nm
6	*m*-Xylene : H_2_O	Very short hexagonal rods with the average length of <300 nm and width of 40–50 nm

a A molar ratio of the metal precursor (ZnCl_2_) to NaOH was maintained at 1 : 5 for each trial. In each case, pH was maintained at the range of 12–13.

**Fig. 3:**
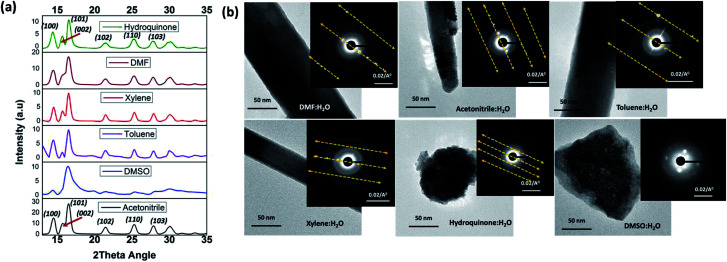
(a) The powder XRD patterns, and (b) the SAED patterns along with the TEM images of ZnO nanostructures prepared in different solvent systems.

The formation of ZnO nanorods, nanoparticles, and random slates like structures with respect to the solvent type can be explained by understanding the solvent-driven shape-controlled crystal growth process. The polarity and chemical nature of the organic solvent in each reaction mixture may act as a selective adhesion surfactant to facilitate the shape-controlled crystal growth. As studied in the past literature, the morphology of the final nanostructure controls by the surface energy and selective adhesion of solvent molecules onto crystallites facets that modulate the crystal growth direction in the nanocrystal unit cell.^35,36^ Since the crystal growth rate is correlated exponentially to the surface energy, surface-energy differences induce much faster growth of the higher surface energy planes and keep the slower growing planes (lower surface energy) as the facets of the product. Comparing the intensities of crystal planes reflections for the crystallites facets of {100}, {001}, and {101} of XRD traces for each solvent system, the preferential crystal growth direction and the morphology of the final nanostructure can be realized. Therefore, we investigated the diffraction patterns for the final products of ZnO nanostructures by acquiring X-ray powder diffraction traces and SAED patterns from the TEM under dark field diffraction mode. As shown in Fig. 3(a), the diffraction patterns of ZnO nanostructures prepared in five of the six solvent systems indexed to hexagonal phase wurtzite crystal structure, and is in good agreement with the previous published literature.^10^ We observed a clear difference in the intensities of [100], [002], and [101] Bragg peaks as well as the peak resolution of the [002] reflection plane in each case. The diffraction pattern obtained for the ZnO nanorods prepared in DMF/water solvent system shows a poorly resolved second order reflection of [001] plane as a shoulder that merges onto the [101] reflection plane. The XRD traces of ZnO nanorods, prepared in the mixtures of acetonitrile, toluene, and xylene with water show a weak intensity peak for the [002] diffraction plane of the first order {001} facet, while maintaining the intensity of the [100] peak by three-fold higher than the intensity of [002] reflection plane. However, the intensity ratios of [100] : [101] diffraction planes for the nanorods prepared in the presence of DMF, acetonitrile, and xylene were 1 : 2, which is slightly higher compared to the intensity ratio of the [100] : [101] peak for the nanorods prepared in the toluene and was found to be 1 : 1.5. The reason for the slight deviation in the intensity ratio could be due to the dimensional difference between thin hexagonal nanorods and wide slates; as observed in Fig. 2. Overall, from these observations, it is evidenced that the presence of poorly resolved second order reflection planes of {001} facets in the XRD patterns of the ZnO nanorods, formed in DMF, acetonitrile, xylene, and toluene could be preferentially bonded to {001} and {002} facets and reduced the crystal growth rate along the second order reflection planes in the [001] direction to yield nanorods. Similarly, in the past literature, it was shown that TiO_2_ nanorods was formed when lauric acid was used and acted as the surfactant that reduces the growth rate along the [001] direction, binding strongly to the {001} facet.^40^

In contrast to the XRD patterns of ZnO nanorods, the XRD pattern obtained for the ZnO nanoparticles exhibits well-resolved first and second order reflection planes for the Bragg peaks of [100], [002], and [101]. The difference in the intensity of these Bragg peaks confirms that the crystal growth rate along the directions of [100], [002], and [101] planes is same. This suggests that there is no preferential adsorption of hydroquinone molecules onto a particular facet over others. In turn, it exhibits equal binding affinity for all facets of crystal lattice. So, the crystals grew into nanoparticles, enabling the crystal growth along all the directions of crystal facets. In contrast, the XRD traces obtained for ZnO nanostructures, prepared in the presence of DMSO show only the well-resolved [101] diffraction plane with a poorly resolved [100] reflection peak. This suggests that DMSO molecules hinder the crystal growth direction along both [100] and [001] faces by selectively binding to both surfaces of {100} and {001} facets.

The SAED pattern of a single nanostructure prepared in each solvent mixture was taken from the TEM under dark field diffraction mode by directing the electron beam at 90° angle to a one of the nanostructure's faces. The SAED patterns collected in this manner are depicted in Fig. 3(b). The SAED patterns viewed along the [001] axis show defect free and single crystalline nanocrystals growth for the nanostructures prepared in all other solvent systems except for the nanostructures prepared in DMSO. The single crystal unit cell diffraction planes are well aligned with the crystal growth directions, revealed from the peak intensities of the respective XRD powder spectra. The SAED patterns for the ZnO nanorods and nanoparticles, show defect free single crystalline pattern with first, second, and third order facets of the nanocrystals' ordering along the [100] diffraction planes. The difference in the well-resolved spacing of higher order reflections at [100] inter-diffraction planes confirms the unit cell packing and unit cell distances of crystalline facets. As observed from the SAED patterns, ZnO nanorods prepared in DMF and toluene exhibit larger nanocrystal unit cell spacing of higher order reflections whereas ZnO nanoparticles, formed in the presence of hydroquinone, show the smallest unit cell spacing in the crystal packing. The crystal growth pattern and nanocrystal packing during the formation of ZnO nanorods in acetonitrile and xylene are alike and exhibit same unit cell spacing among the higher order reflections. The SAED pattern obtained for ZnO nanostructures formed in DMSO (also see Fig. S2†) reveals weak polycrystalline pattern with poorly resolved nanocrystals ordering along [100] plane, confirming truncated crystal facets with polycrystallinity to amorphous.

### X-ray photoelectron spectroscopy analysis of different morphologies of ZnO nanostructures

**Fig. 4:**
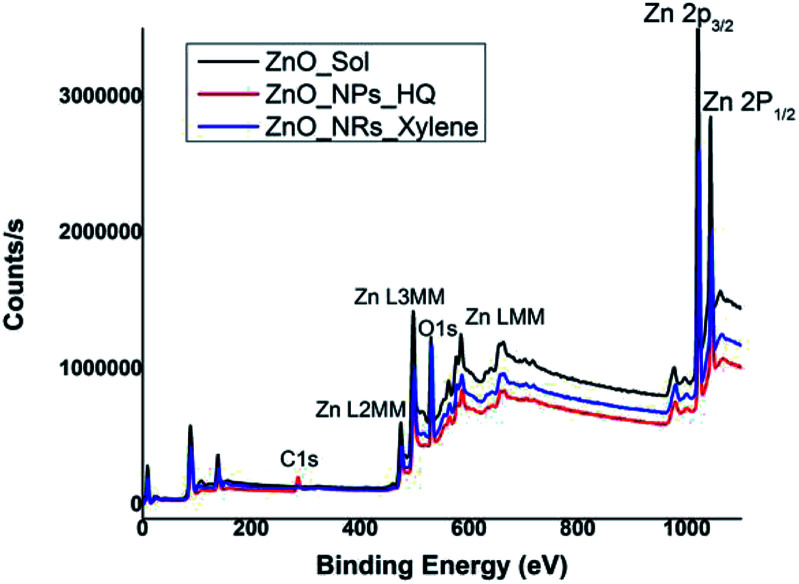
XPS wide survey spectra of ZnO sol, nanorods (formed in *m*-xylene), and nanoparticles (formed in hydroquinone).

In order to correlate the selective solvent adhesion onto crystal facets and the shape-controlled crystal growth process that yields ZnO nanostructures with either nanorods or nanoparticles, X-ray photoelectron spectroscopy (XPS) analysis was conducted. The XPS wide survey spectra obtained for ZnO nanorods, nanoparticles, and sol, are depicted in Fig. 4. In all three cases, the atomic orbital bonding states of Zn and O peaks were detected and confirms the presence of strong Zn–O bonds on the nanostructure surface. The Zn peaks observed at 1044 eV (±2.10 eV), 1020 eV (±2.20 eV), 498 eV (±0.10), and 475 eV (±0.15 eV) were attributed to the chemical states of the Zn species, which include higher binding energy states for Zn 2p_1/2_ and Zn 2p_3/2_ of Zn–O bonds, and lower binding energy states for interstitial zinc (Zn_i_) respectively.^49^ The carbon 1s peak is only noticeable in the XPS survey spectrum of ZnO nanoparticles, prepared in hydroquinone/water solvent system. The presence of carbon 1s peak in nanoparticles suggests that there is a considerable concentration of organic solvent molecules adsorb onto the nanoparticle surface. This supports our prediction of adsorbing hydroquinone molecules onto all the crystal facets of the lattice structure without any selectivity towards a particular crystal facet.

The Table S1† summarizes binding energy (BE) for the core levels Zn 2p, O 1s, C1s and their corresponding full-width at half maximum (FWHM) along with the respective %atomic concentrations for two types of ZnO nanostructures and sol. The C 1s atomic concentration for nanoparticles is twenty times higher than that of ZnO sol and five times higher than that of nanorods. Overall, with the increase in carbon and oxygen atomic concentrations and a gradual decrease in Zn atomic concentration clearly evidence that the presence of adsorbed solvent molecules on crystallites facets that affect in modulating the crystal growth direction in the nanocrystal unit cell. As we discussed in the morphology analysis section, it is clear that *m*-xylene prefers binding only onto a particular facet, hindering the crystal growth along the respective facet, yielding rod shape nanostructures. Whereas hydroquinone shows equal binding affinity onto all the facets of the crystal lattice and adsorb solvent molecules onto all the facets, yielding spherical shape nanostructures.

**Fig. 5:**
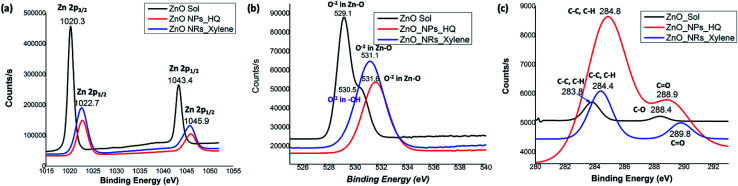
The comparison graphs of orbital binding energy states of high resolution XPS spectra of: (a) Zn 2p region, (b) oxygen 1s core-level, and (c) carbon 1s state for ZnO sol, nanorods, and nanoparticles.

The noticeable differences in the binding energy spectra of Zn 2p, O 1s, and C 1s of ZnO sol, nanorods, and nanoparticles reveal the chemical environment interaction between solvent molecules and the surface atoms of the crystal facets. Fig. 5 demonstrates the comparison of orbital binding energy states of high resolution XPS spectra of the Zn 2p region, O 1s core-level, and C 1s for ZnO sol, nanorods, and nanoparticles. The binding energies of the Zn 2p components of sol are slightly lower than the binding energies of the Zn 2p components of nanorods and nanoparticles (Fig. 5(a)). The low binding energy of Zn 2p in sol evidences that the chemical environment interaction of Zn–O bonding surfaces of sol could be different from the chemical environment interactions of nanorods and nanoparticles. This may also be due to the difference in surface morphology and crystal size in nanocrystals compared to nanorods and nanoparticles.^50^ Surprisingly, the binding energy peaks of the Zn 2p components for ZnO nanorods and nanoparticles are located at the same positions. This suggests that the nature of chemical interactions in both rods and particles are attributed to strong Zn–O bonding interactions. In overall, Zn 2p components binding energy spectral traces indicate that the chemical valence of Zn at the surface of nanostructure morphologies and sol is Zn^2+^ oxidation state. The binding energy difference between the Zn 2p_1/2_ and Zn 2p_3/2_ is 23 eV for ZnO nanostructures and ZnO sol.

The O 1s spectrum for ZnO sol in Fig. 5(b) shows a sharp peak at 529.1 eV and a shoulder peak at 530.5 eV. The lower energy peak is attributed to O^2−^ in Zn–O bonding of the wurtzite structure of ZnO.^51^ The higher binding energy (530.5 eV) is usually related to OH group absorbed onto the surface of the ZnO nanoparticles.^50^ However in our case, sol usually contains a mixture of ZnO and Zn(OH)_2_. It is expected to have OH groups on nanocrystal surface. The O 1s spectra of nanorods and nanoparticles, shown in Fig. 5(b), show only O^−2^ in Zn–O bonding with a shift in binding energy of 2 eV and 2.5 eV, respectively, compared to the binding energy peak of O^−2^ in ZnO sol. The peaks positions at 531.1 eV and 531.6 eV in nanorods and nanoparticles are in good agreement with the previous work of ZnO nanorods and nanoparticles.^50,51^ The C 1s spectra of sols, nanorods, and nanoparticles show three different binding energy peaks, which are uniquely characteristic to each morphology of ZnO nanostructures. The C 1s spectrum of ZnO sol shows two very weak intensity peaks at 283.8 eV and 288.4 eV, reflecting C–C/C–H and C–O bonding. In our case, these weak C–C/C–H and C–O bonding energies are attributed to traces of ethanol molecules adsorbed onto the nanocrystal surface during the washing of centrifuged powder form of ZnO sol. However, the binding energy peak for C–C/C–H in nanorods and nanoparticles are clearly visible and slightly shifted to yield binding energy of 284.4 eV and 284.8 eV respectively. In nanoparticles, the intensity counts for the C–C/C–H bonding binding energy is significantly higher than nanorods and confirms the presence of adsorbed organic molecules onto the nanoparticle surface. The higher binding energy peaks at 289.8 eV (weak peak) and 288.9 eV (strong peak) in nanorods and nanoparticles, respectively, are attributed to carbonyl (C=O) bonds. The presence of a strong peak for carbonyl bonds in nanoparticles further confirms that the adsorbed hydroquinone molecules on nanoparticle surface.

### Optical properties and band gap analysis

**Fig. 6:**
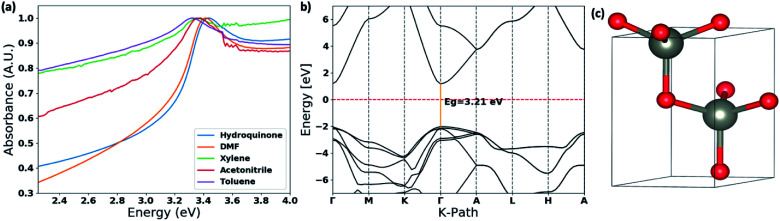
(a) Thin film UV-visible spectra of the ZnO nanostructures with respect to the solvent type; (b) the calculated band structure for ZnO wurtzite hexagonal lattice structure obtained by matching with the simulated XRD spectra and the experimental XRD traces (Fig. S3†). The energy of the valence-band maximum (VBM) was set to zero and used Hubbard U+DFT hybrid function for the calculation; and (c) respective ZnO crystal structure extracted from the crystallography data base to compute the band structure.

The thin films UV-visible spectra of ZnO nanostructures, prepared in different solvent systems, were collected and is depicted in Fig. 6(a) and S4.† The optical band gaps were calculated from the on-set of the spectra in each case. The deviation of the optical band gap with respect to the different nanostructures formed in each solvent type were calculated by collecting UV-visible thin film spectra of 5–7 samples prepared from each solvent type. Depending on the nanostructure morphology, there is a noticeable shift in the absorption maxima. The photo-absorption energy (*hv*) of the absorption maximum is ranged from 3.29 eV to 3.58 eV with respect to the ZnO nanostructures formed in different solvent systems.

The comparison in the energy absorption maxima and optical band gaps of the ZnO nanostructures formed in different solvent systems are summarized in Table 2. The ZnO nanorods and nanoparticles formed in DMF and hydroquinone solvent systems show maximum absorption energies at 3.42 eV and 3.58 eV respectively. The nanoparticles' optical band gap, calculated from the UV on-set was ranged from 3.33–3.41 eV, which is considerably higher than the optical band gap range of 3.16–3.24 eV for nanorods made in DMF solvent system. The ZnO nanorods prepared in acetonitrile, toluene, and xylene solvent systems exhibit the absorption maxima ranging from 3.30–3.39 eV with the lowest optical band gap of 3.10 eV for nanorods formed in acetonitrile. The optical band gaps of the ZnO nanorods formed in toluene and xylene are 3.22 ± 0.10 and 3.19 ± 0.04 respectively. The changes in the optical band gaps with respect to the ZnO nanostructure morphology further confirms that nanostructure crystallinity, crystal growth facets, and crystal grain size lead to the effective band gap of nanostructured ZnO smaller than its bulk value of 3.37 eV.

**Table 2:** The energy absorption maxima and optical band gaps of the ZnO nanostructures formed with respect to solvent systems

Solvent system	Energy (*hv*) of the absorption maximum (eV)	Optical band gap (eV)
DMF : H_2_O	3.40 ± 0.02	3.20 ± 0.04
Acetonitrile : H_2_O	3.36 ± 0.03	3.15 ± 0.05
Toluene : H_2_O	3.33 ± 0.03	3.22 ± 0.10
Hydroquinone : H_2_O	3.50 ± 0.08	3.33 ± 0.04
*m*-Xylene : H_2_O	3.34 ± 0.04	3.19 ± 0.04

The optical band gap of ZnO nanorods formed in DMF solvent systems was compared with the theoretical band gap computed from the respective ZnO crystal structure, which was extracted from a crystallographic data base, and followed by adjusting the unit cell parameters comparing the simulated XRD pattern of the extracted ZnO crystal structure with the experimental XRD traces of ZnO nanorods. The theoretical band structure was computed using the plane-wave form of Density Functional Theory (DFT) implemented in the open source Quantum ESPRESSO (QE) suite. Ultra-soft pseudopotentials created with low-density approximation (LDA) functions were used and were obtained from QE. The band gap calculated using only DFT was severely underestimated, forcing a change to DFT+U; this scheme uses an additional Hubbard U potential for each element to correct band overlap discrepancies. The potentials for zinc and oxygen to correct the large discrepancy were based on literature, 12 eV and 6.5 eV respectively. The potentials were applied to the 3d orbitals of the Zn atoms and the 2p orbitals of the O atoms.^52,53^ With this method, we were able to compute the corrected band gap to be 3.29 eV (Fig. 6(b)), which is in good agreement with the optical band gap of ZnO nanorods formed in the DMF solvent system.

### Electrical properties

**Fig. 7:**
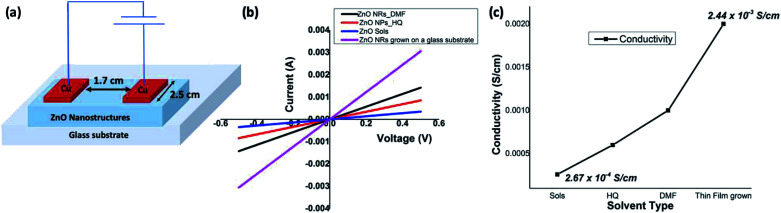
(a) A schematic diagram of a test device used for *I*–*V* characterization; (b) *I*–*V* graphs of ZnO nanostructures; and (c) the electrical conductivities of solution formed nanorods and nanoparticles comparing with the electrical conductivity of sols and solvothermal grown nanorods from ZnO sols-casted glass substrate.

ZnO nanorods, formed in DMF/water and ZnO nanoparticles, formed in hydroquinone/water, along with ZnO sol and solvothermal grown ZnO nanorods were chosen to evaluate and compare the electrical properties of ZnO at nanoscale. The electrical conductivities were measured using a four-probe technique and a device configuration of a fabricated test device is depicted in Fig. 7(a). The detailed experimental set up is described in the Experimental section. The average electrical conductivity of each morphology of ZnO nanostructures was calculated from the slopes of the multiple IV curves. The *I*–*V* curves of nanorods, nanoparticles, and sol are shown in Fig. 7(b) along with ZnO nanorods directly grown from a ZnO sol-casted thin film substrate using the solvothermal process. The ZnO nanorods, formed in DMF/water solvent mixture, exhibits an average electrical conductivity of 1.14 × 10^−3^ S cm^−1^ (±0.01), which is almost one order of magnitude higher than the electrical conductivity of ZnO nanoparticles and ZnO sol. The average electrical conductivity of ZnO nanoparticles and sol were found to be 6.65 × 10^−4^ S cm^−1^ (±0.06), and 2.67 × 10^−4^ S cm^−1^ (±0.13), respectively. The significant increase in the electrical conductivity of ZnO nanorods prepared in DMF solvent system further agrees with its narrow optical band gap, compared to the optical band gap of ZnO nanoparticles. As shown in Fig. 7(c), the electrical conductivity of ZnO nanostructures gradually increases with respect to nanocrystal size, shape, crystallinity, and nanostructure orientation. In the case of sol, we observed the lowest electrical conductivity due to non-ripened nanocrystals. The nanorods grown from thin-film casted sols shows the highest electrical conductivity (2.44 × 10^−3^ ± 0.005 S cm^−1^) as a result of crystal shape, defect free crystallinity, and nanorods orientation on the glass substrate compare to the solution-processed ZnO nanorods thin films (see Fig. S5†). In overall, the electrical conductivities of solution-processed thin films of ZnO nanorods and solvothermal grown ZnO nanorods are in good agreement with a typical ZnO field effect transistor's peak conductivity of 1.25 × 10^−3^ S cm^−1^.^10^

## Experimental

### Materials

Sodium hydroxide (98% purity), and zinc chloride (97% purity), were obtained from Aldrich Chemicals. Anhydrous dimethyl formamide, anhydrous dimethyl sulfoxide, *meta*-xylene, acetonitrile, hydroquinone, and toluene were used as received without any purification otherwise specified.

### Characterization

The powder XRD analysis was conducted using Mo Ka radiation (40 kV, 40 mA, *k* = 0.7093 Å) with a speed of 60 s on the X-ray diffractometer (XRD, Agilent technologies Gemini). The morphology and the size of ZnO nanostructures were analysed using transmission electron microscopy (TEM Carl Zeiss Libra 120) at 120 keV and scanning electron microscopy (Zeiss Auriga FIB/FESEM). The Atomic concentrations and binding energies of all the elements present in ZnO nanostructures with different morphologies were obtained from X-ray photoelectron spectroscopy (XPS) using XPS-Escalab Xi+ Thermo Scientific electron spectrometer. The binding energies were corrected for the charge shift using the C 1s peak of graphitic carbon (BE = 284.6 eV) as a reference. The optical properties were determined using ultraviolet-visible spectroscopy (UV-vis spectroscopy, Varian Cary 6000i). The electrical properties were determined using Keithley source meter controlled by a Photo Emission TEC. INC (PET) *I*–*V* test system. E-beam evaporation (Kurt Lesker PVD 75 e-beam evaporator) was used to deposit Cu (100 nm). Thin films were prepared by spin coating the sample onto ozone/UV treated (Bio Force UV/Ozone Pro Cleaner) glass coverslips and silicon substrates.

### Typical sol–gel synthesis procedure for the preparation of ZnO nanostructures

In a typical procedure, the synthesis of ZnO nanostructures was performed using the wet chemical synthesis in an aqueous-based solvent mixture by maintaining the molar ratio of zinc chloride precursor to sodium hydroxide at 1 : 5 mmol. First, zinc chloride (0.21 g) was dissolved in a selected organic solvent (∼30 mL) and a solution of NaOH (0.40 g in 5 mL water) was added into the reaction mixture. The solution was initially stirred at room temperature about 15 minutes and continued stirring another 60 minutes at 80 °C. Then, the reaction flask was sealed and transferred onto a sand bath and continued heating at 80 °C for 24 hours with no stirring. The white solid was collected by centrifugation and repeated washing with water to remove salt, and unreacted base and the precursor. The white powder of ZnO nanostructures yielded (∼110 mg) was re-suspended in water (∼20 mL) with sonication as necessary. The suspension was drop casted on a cleaned silicon substrate and imaged under SEM. The UV-visible spectra of ZnO nanostructures were obtained in water/ethanol mixture. The solvothermal grown ZnO nanorods on a glass substrate was obtained by spin coating the solution of sols on a cleaned glass substrate followed by placing the substrate face-up in an aqueous mixture (DMF and water) of zinc chloride precursor and the base at 80 °C for 24 hours with no stirring.

### Electrical conductivity measurements

Electrical conductivities of all test devices were measured using copper as a common electrode using four-probe method. For this purpose, spin-coated thin films of ZnO nanostructures only devices were fabricated. Glass substrates were cleaned prior to device fabrication. First, substrates were washed with Isopropyl Alcohol in an ultrasonic bath for ten minutes, followed by washing with soap solution. Substrates were then rinsed in deionized water and treated with a mixture of ammonium hydroxide and hydrogen peroxide for 15 minutes at 50–70 °C. After the above treatment, the plates were again washed with deionized water under ultrasonication for 15 minutes and blown dry with dry nitrogen. The cleaned glass substrates were subject to UV cleaning for 35 min prior to the deposition of the sample. A solution of ZnO nanostructures (25 mg in 1 mL NMP) was spin coated on the substrate in nitrogen atmosphere at the rate of 1400 rpm for 10 seconds to spread, then 3000 rpm for 50 seconds to dry followed by annealing at 120 °C for 5 min. This process was repeated six times using 70 μL of solution followed by vacuum drying under nitrogen atmosphere. The test devices prepared in this manner were subjected to the deposition of the cathode (100 nm thick Cu layer) on top of the active layer using PVD e-beam deposition and transferred into a nitrogen filled sealed chamber to prevent any oxidation of the copper layer and transferred into a nitrogen filled sealed chamber to prevent any oxidation of the copper layer. *I*–*V* measurements were conducted immediately after the deposition of the cathode. A schematic diagram of a test device is depicted in Fig. 7(a). For *I*–*V* measurements, the channel length was kept constant at 1.7 cm while the active cell area maintained at 4.25 cm^2^. Conductance of samples was calculated from the slope of the ohmic region of *I*–*V* curves and conductivities were obtained from;
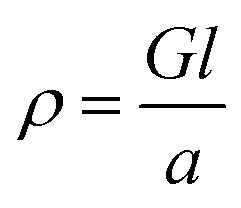
where *ρ* is conductivity, *G* is conductance, and *l* and *a* are channel length and area respectively. Each sample was tested multiple times to ensure reproducibility of the results.

### The method used for the theoretical band structure calculation

The theoretical band gap and the respective band structure were computed using the open source Quantum ESPRESSO (QE) suite. QE uses plane-waves to find solutions to a specific density functional theory (DFT) setup. The computations were done using Ultra-soft pseudopotentials created with LDA functions. Based on an optimization calculation the global cut-off energy for the calculation was set at 100 Ry. The ZnO wurtzite CIF structure file used for the computations was from the Materials Project with the ID: mp-2133. The visualization software VESTA was used to compute the theoretical XRD from the CIF file. With both the experimental and theoretical XRD, the unit cell's *a*, *b* and *c* values were changed slightly to make sure the computed material matched the experimental material. The unit cell values used were *a* = *b* = 3.22 A and *c* = 5.16 A.

## Conclusions

In summary, the one-pot wet-chemical method that follows a versatile sol–gel synthesis, combined with low-temperature solvothermal process, allows us to make highly crystalline ZnO nanostructures with controlled-morphologies. The crystal growth process driven by the solvent polarity, augments the crystal growth-oriented attachment mechanism. As evidenced by XRD and XPS analysis, the growth of crystal facets is governed by the relative surface energy of crystal facets and selective adhesion of solvents (act as surfactant in our case) onto crystal facets. The morphologies of the ZnO nanostructures formed in different solvent systems were either nanorods, or nanoparticles, or nanoslates. The nanorods and nanoparticles show highly ordered, defect free wurtzite crystalline structures. The effective optical band gap of ZnO nanostructures with respect to their morphology was able to tailor within the range of 3.37 eV to 3.10 eV by controlling the crystallites size and shape. The enhanced electrical conductivities further suggest the correlation of optical and electrical properties to crystal growth shape, size, and orientation, which easily could be tuned through this synthesis approach of combined sol–gel and solvothermal crystal growth process. Thus, this sol–gel synthesis, combined with solvent-driven solvothermal method offers simple, versatile, and environmentally friendly wet-chemical method to make band gap-engineered metal oxide nanostructures, with enhanced optical and electrical properties, from a wide variety of metal cation precursors.

## Conflicts of interest

There are no conflicts to declare.

## Supplementary Material

RA-009-C9RA02091H-s001
