# Supplementary material for: Band gap engineered zinc oxide nanostructures via a sol–gel synthesis of solvent driven shape-controlled crystal growth
Source: RSC Adv. 2019 May 10;9(26):14638–48. doi: 10.1039/c9ra02091h (PMC9064155; doi:10.1039/c9ra02091h)
Supplement: RA-009-C9RA02091H-s001 [file RA-009-C9RA02091H-s001.pdf]

Electronic Supplementary Material (ESI) for RSC Advances.
This journal is © The Royal Society of Chemistry 2019

Band Gap Engineered Zinc Oxide Nanostructures via a Sol-Gel Synthesis of Solvent Driven Shape-Controlled Crystal Growth

Klinton Davis,[a] Ryan Yarbrough,[a] Michael Froeschle, [a] Jamel White, [a] and Hemali Rathnayake[a]*

[a]Department of Nanoscience, The Joint School of Nanoscience and Nanoengineering, University of North Carolina at Greensboro, Greensboro, NC 27401

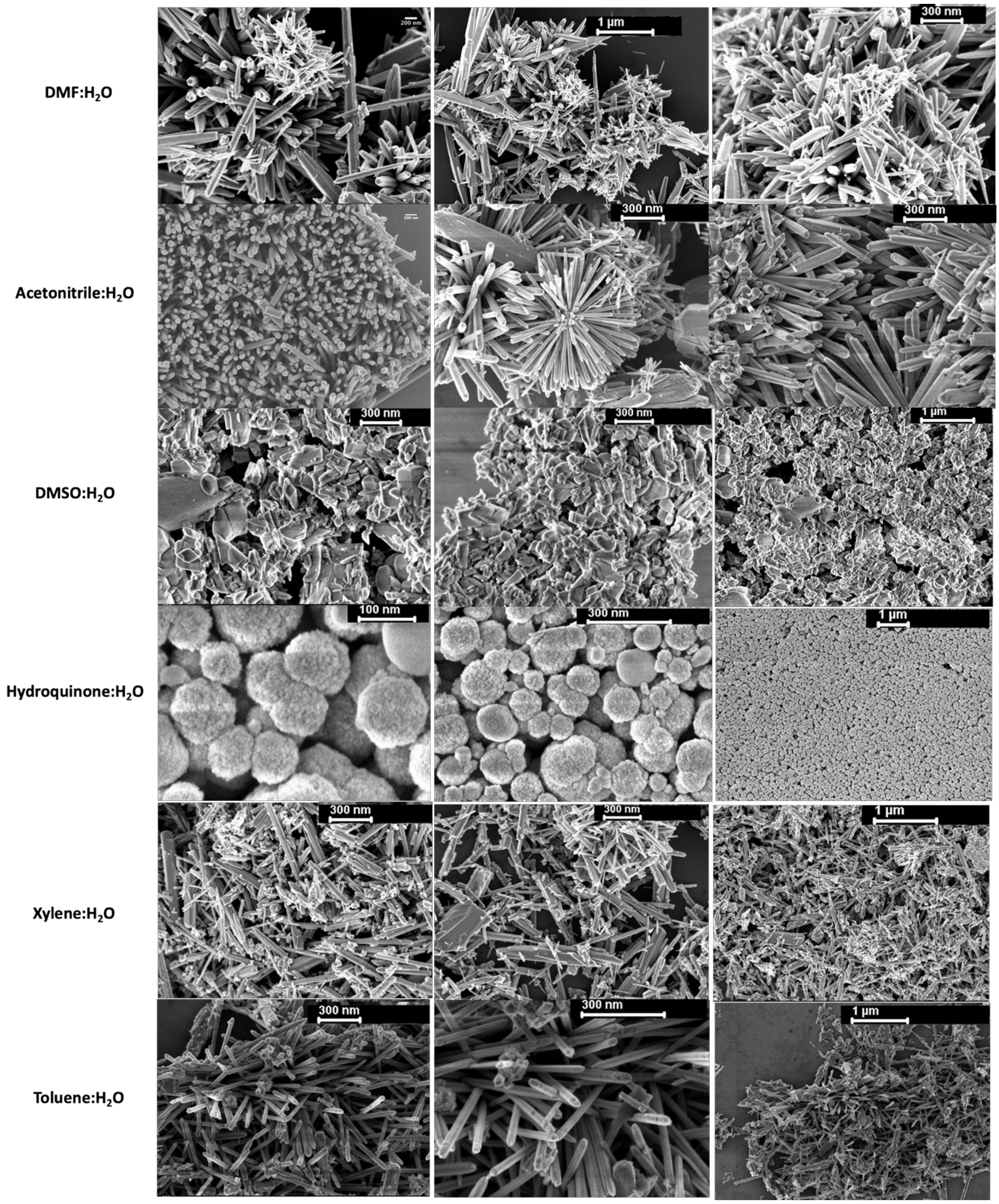

Figure S1: SEM images of ZnO nanostructures formed from each different solvent system.

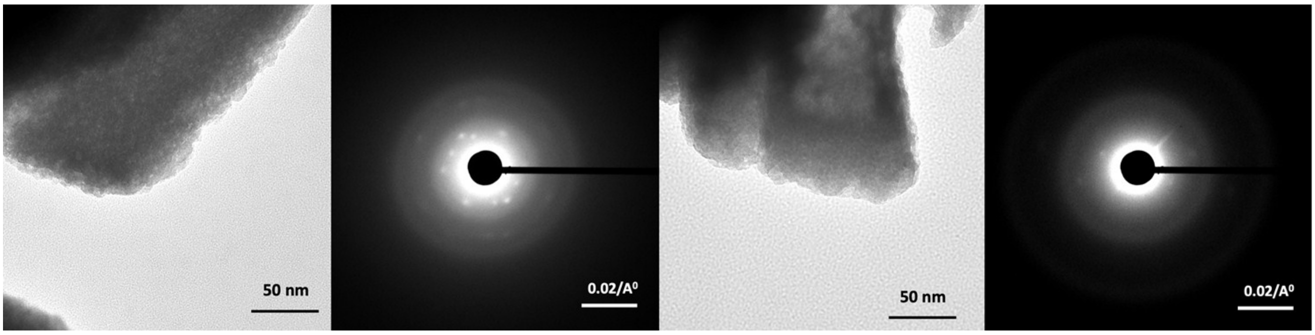

Figure S2: TEM images and SAED patterns of ZnO nanoslates formed in DMSO:water solvent system.

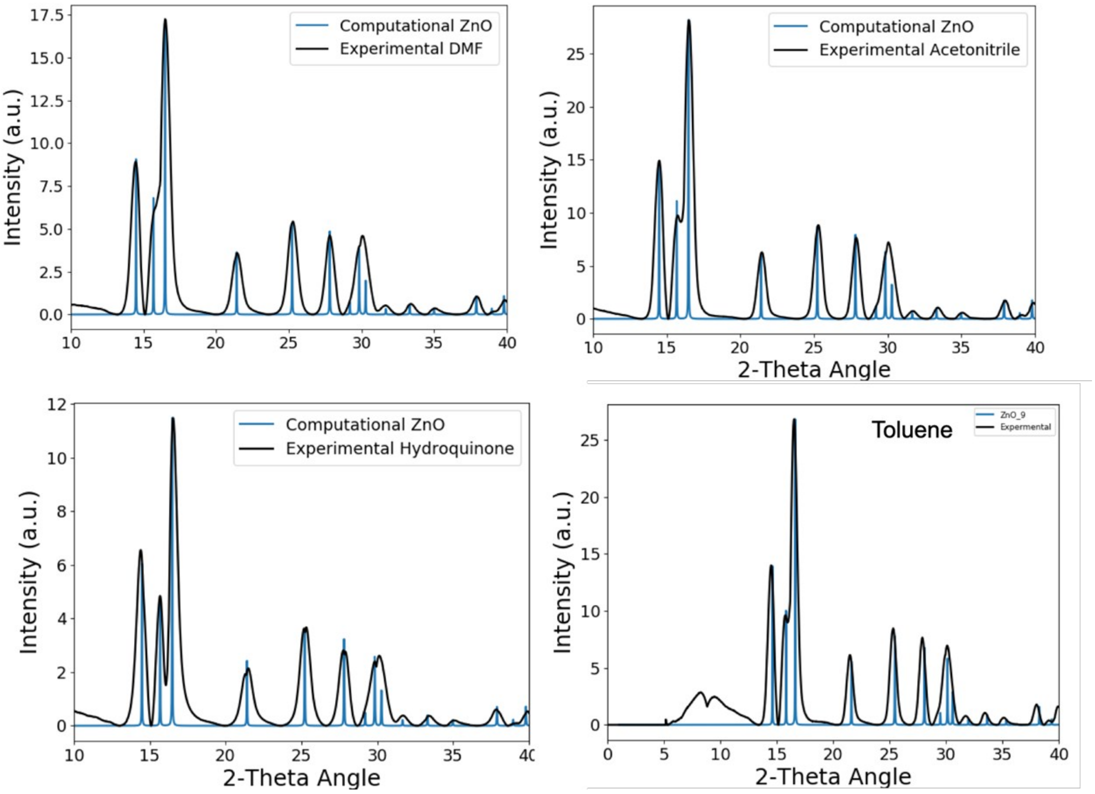

Figure S3: Experimental XRD traces and simulated XRD spectra of ZnO nanostructures with respect to the solvent type.

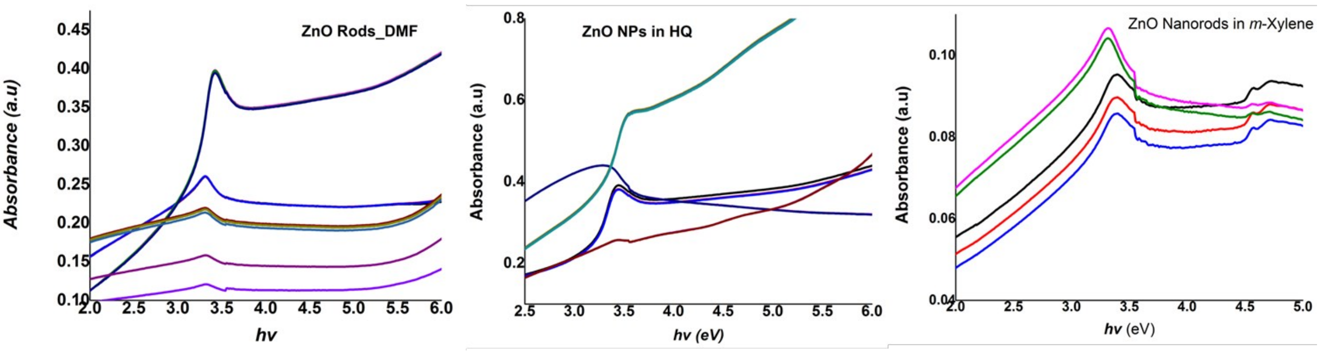

Figure S4: UV-vis spectral traces, collected from multiple sample trials of ZnO nanostructures-casted on thin films.

Table S1: XPS analysis of ZnO nanostructures for sol, nanorods, and nanoparticle morphologies.

Morphology	Peak	Position BE (eV)± 0.10 eV	FWHM (eV)± 0.20 eV	Atomic Con. (%)
ZnO sol (nanocrystals)	Zn 2p $_{½}$; Zn 2p $_{3/2}$	1043.4; 1020.3	1.44	51.51
	O 1s	529.1; 530.5	1.18	47.85
	C 1s	283.8; 288.4	1.27	1.26
ZnO NRs (m-Xylene)	Zn 2p $_{1/2}$; Zn 2p $_{3/2}$	1045.9; 1022.7	4.76	35.13
	O 1s	531.1	4.31	50.72
	C 1s	284.4; 289.8	3.93	4.80
ZnO NPs (Hydroquinone)	Zn 2p $_{½}$; Zn 2p $_{3/2}$	1045.9; 1022.7	4.62	26.88
	O 1s	531.6	4.41	45.21
	C 1s	284.8; 288.9	5.65	27.90
